# The Micronutrient Status of Children Aged 24–60 Months Living in Rural Disaster Areas One Year after the Wenchuan Earthquake

**DOI:** 10.1371/journal.pone.0088444

**Published:** 2014-02-12

**Authors:** Caixia Dong, Pengfei Ge, Xiaolan Ren, Xianfeng Zhao, Jie Wang, Haoqiang Fan, Shi-an Yin

**Affiliations:** 1 Department of Chronic disease, Gansu Center for Disease Control and Prevention, Lanzhou City, Gansu Province, China; 2 Department of Maternal and Child Nutrition, National Institute of Nutrition and Food Safety, Chinese Center for Disease Control and Prevention, Beijing, China; Old Dominion University, United States of America

## Abstract

**Objective:**

In order to evaluate micronutrient status of children aged 24–60 months living in rural disaster areas after one year of the earthquake in Wenchuan.

**Design:**

After one year of Wenchuan Earthquake, using PPS sampling methods, a total of 270 children from six-randomized townships near seismic center, in each township sample size consisted of 30 to 50 children, were sampled for evaluating Z-score of children's growth status, anemia prevalence, vitamin A, vitamin D, vitamin B_12_, folic acid status. Final sample consisted of 152 boys and 118 girls, and blood samples were drawn from 206 children.

**Results:**

The stunting (HAZ<2SD) and wasting (WHZ<2SD) were respectively 14.7% and 0.7%. Prevalence of anemia was 17.3% and percentage of iron deficiency was 45.7%. The prevalence of vitamin A deficiency and marginal deficiency was 15.4% and 30.3%, respectively. The sum of vitamin D deficiency and marginal deficiency was near 90%. Percentages of Zinc deficiency including marginal and deficiency were 65.5%. Percentages of vitamin B_12_ marginal and deficiency were 8.6% and 10.6% and the prevalence of marginal deficiency was significantly higher in boys than in girls. Folic acid deficiency was not found in surveyed children using serum folic acid level <16.9 ng/ml.

**Conclusion:**

In areas affected by Earthquake, preschool children had higher stunting prevalence and a relatively higher prevalence suffered from micronutrient deficiencies, including iron-deficiency anemia, and deficiencies of iron, zinc, vitamin A and vitamin B_12_.

## Introduction

Earthquake in Wenchuan occurred on May 12, 2008 in Sichuan, Gansu and Shanxi provinces of China severely destroyed the infrastructure and social networks on which people usually depend, and caused that the living conditions had been dramatically changed for entire communities and normal lives, especially resulted in that the food supply system was severely damaged or even completely stopped and families were left without shelter and the basic necessities of life.

During such situations, major food shortages have been showed to be a primary feature in such areas. Food supplies (A general food basket) provided by government could meet the basic energy and nutrient requirements for adults, the per capita cereal supply was 500 grams per day which could provide about 1730 kcal/per day, and if additional energy from the non-staple food consumption (vegetables and fruits), a general food basket could provide 2100 kcal energy per person per day that met the needs for the basic energy, protein, but most of micronutrient requirements could not be met. However, for a special physiological state, such as preschool children aged 24–60 months have been identified as the most nutritionally vulnerable group in these situation [1]–[3], these foods would be difficult to meet the needs to a variety of micronutrients so that these populations would be at high risk suffered from nutritional deficiencies due to a low intake of animal source of foods [3], [4], especially micronutrients such vitamin A and vitamin D, iron, zinc, vitamin B_12_ which would be most likely to be deficiency in children. There are some evidences that these micronutrients have specific critical roles in children's growth and brain nutrition [5]–[8].

The primary objective of this survey was to assess growth status, the prevalence of anemia and the other micronutrient deficiencies (including vitamin A and vitamin D, zinc, vitamin B_12_ and folic acid) in the children aged from 24 to 60 months living in rural disaster areas one year after the Earthquake, and to provide the basic evidence for making strategy on nutrition intervention for such as areas.

## Materials and Methods

All survey sites located in high mountain and inaccessible areas, and were near seismic center of Wenchuan Earthquake on May 12, 2008. The life and property losses of local residents were very severe. The present surveys were approved by the ethic committee of Gansu Province Center for Disease Control and Prevention, and National Institute of Nutrition and Food Safety of Chinese Center for Disease Control and Prevention, and written informed consent was obtained from all parents or guardians.

### Design and sampling

On April 2009 (about one year after the Earthquake), a nutritional survey was conducted in rural disaster areas near seismic center of Earthquake, which was coordinated by County and Provincial Center for Disease Control and Prevention organized by National Institute of Nutrition and Food Safety of Chinese Center for Disease Control and Prevention. Using PPS sampling methods, a total of 300 children from six-randomized townships near seismic center, in each township sample size consisted of 30 to 50 children aged 24–60 months, were sampled for evaluating Z-score of children's growth status, anemia prevalence, vitamin A, vitamin D, vitamin B_12_ and folic acid status.

### Data collection

A questionnaire was answered by the children's mothers or other caregivers. The body weight and height were measured to evaluate the low body weight and growth retardation. A platform weighing scale (TC100KA, 0–100 kg of capacity and 10 g of accuracy, Huatec company, China) and height scale (YSC-2, 0.1 cm of accuracy, Beijing Guowangxingda weight Scale Company, China) were used to measure body weight and height of children wearing only underwear. Before each measurement, the weighing and height scales were checked with the calibrated weight material and the nurses should be trained and evaluated.

### Blood sampling and biochemical analyses

A fasting blood sample (3–5 ml) was drawn from an antecubital vein by local hospital technicians in the morning. The blood specimens were collected in redcap serum separation tubes and immediately wrapped with aluminum foil and kept cold about 60 minutes, centrifuged at 2500–3000 rpm for 10 min at room temperature, and then the serum fraction was collected and placed in a refrigerator at −20∼−30°C within 5 days in the field site. After finished the field work, all serum samples in frozen state were transferred into Beijing by air and stored in −80°C until they were analyzed.

Hemoglobin concentration in whole blood was assayed using the hemocue (HB 301, HemoCue AB, Angelholm, Sweden) in the field sites. Anemia was defined as a hemoglobin level of <110 g/L, for those sites with altitude over 1000 meters, the anemia prevalence was corrected using WHO recommended altitude formula.

Analyses of the other micronutrients in serum were organized and performed by National Institute of Nutrition and Food Safety of Chinese Center for Disease Control and Prevention. Serum retinol was assessed by using modified HPLC method (Waters 600E, US) [9]; serum 25-OH-D_3_, vitamin B_12_, folic acid and ferritin were determined by using commercial radioimmunoassay kits (25-OH-D_3_, Dria Sorin, US; vitamin B_12_ and folic acid, MP Biomedicals, US; ferritin, Northern Institute of Biotechnology, China); serum zinc was determined by using flame atomic absorption spectrometry (VARIAN, US).

### Bias

Because the surveyed sites located in the high and rugged mountain areas and the living place was much decentralized and inaccessible areas, the sample size at each selected site was not inconsistent by the reasons that some of the children could not catch up with the survey due to the bad weather and raining, or remote and inaccessible transportation from their living areas, which could lead to the potential bias.

### Statistical analysis

SAS software (version 9.1; SAS Institute Inc, Cary, NC) was used for the statistical analysis. The general linear models were used to compare the means of body height and weight, height-for-age z score (HAZ), weight-for-age z score (WAZ), weight-for-height z score (WHZ), growth status was evaluated by WHO Child Growth Standard (2006), and malnutrition was defined as a z score less than −2. Data were expressed as mean±SD.

## Results

### Growth and development status and z-scores of children

The sample consisted of 152 boys (56.3%) and 118 girls (43.7%), and blood samples were drawn from 206 children. The average age (m±SD) of three age group was 29.1±3.5, 42.2±3.7 and 53.7±2.8 for boys, and 29.4±3.3, 42.6±3.5 and 53.2±2.6 for girls, there was no significant difference between boys and girls. Average Z-scores of children in each age group were shown in table 1. The stunting (HAZ<2SD) and wasting (WHZ<2SD) were respectively 14.7% and 0.7% without gender difference.

**Table 1:** Average Z-scores of children^1^.

Age (m)	Boys				Girls			
	n	WAL/WHZ^2^	HAZ^3^	WAZ^4^	n	WAL/WHZ	HAZ	WAZ
24–36	56	0.68±0.92^a^	−0.86±1.15^a^	0.03±0.96^a^	44	0.48±0.63^a^	−0.86±1.00^a^	−0.10±0.73^a^
37–48	51	0.24±0.93^b^	−0.91±1.29^a^	−0.37±0.82^b^	40	0.23±0.98^a^	−0.45±1.26^b^	−0.10±0.93^ab^
49–60	45	0.29±0.96^b^	−0.01±1.00^a^	−0.42±0.83^b^	34	0.18±0.82^a^	−0.99±0.94^ab^	−0.48±0.83^b^

1 The results were expressed as mean±SD and means in the same column with different superscripts a–c were significantly different as a result of different age at the *P*<0.05 level.

2 WAL/WLZ Z-score of weight for length for infants and young children less than 24 months.

3 WHL Z-score of weight for height for children equal or over 24 months, Z-score of weight for height.

4 HAZ, Z-score of height for age; WAZ, Z-score of weight for age.

### Hemoglobin concentration and anemia and iron-deficiency prevalence of children

The hemoglobin concentration, anemia prevalence and iron status of children were shown in Table 2. Based on Hb<110 g/L recommended by World Health Organization, the average prevalence of anemia was 17.3%. Average serum ferritin levels were 21.9±23.9 µg/L and percentage of iron deficiency was 45.7% using 15 µg/L as cut-off point, and the highest iron deficiency was the children aged 24–36 months. The ferritin level was increased with the age and then iron deficiency was decreased with the age. There was no gender difference in hemoglobin concentration, anemia, and iron deficient prevalence.

**Table 2:** Hemoglobin and ferritin concentrations and prevalence of anemia and iron deficiency of children^1^.

Age (m)	Boys						Girls					
	n	Hb g/L	Anemia %	Serum ferritin			n	Hb g/L	Anemia %	Serum ferritin		
				n	Content ng/ml	Fe-deficient %				n	Content ng/ml	Fe-deficient %
24–36	56	123.0±10.5^a^	12.1^a^	34	17.6±13.9^a^	58.8^a^	44	121.1±11.2^a^	21.3^a^	26	17.0±13.7^a^	52.0^a^
37–48	50	120.4±11.9^a^	18.0^a^	42	20.8±16.0^a^	41.0^ab^	39	118.8±12.5^a^	30.8^ab^	27	21.2±17.7^a^	48.0^a^
49–60	45	122.0±10.4^a^	14.3^a^	37	25.6±19.8^a^	35.1^b^	34	124.5±9.8^a^	8.6^b^	30	27.7±47.8^a^	40.0^a^

1 The results were expressed as mean±SD and means in the same column with different superscripts a–c were significantly different as a result of different age at the *P*<0.05 level.

### Vitamin A and vitamin D status and deficiency prevalence of children

The levels of serum retinol and 25-OH-D_3_ and deficiency prevalence of vitamin A and vitamin D of children were shown in Table 3. Vitamin A status was evaluated by serum retinol level as follow: marginal deficiency, serum retinol<1.05 µmol/L; deficiency, serum retinol<0.7 µmol/L. The prevalence of vitamin A deficiency and marginal deficiency was 15.4% and 30.3%, respectively. The trend on vitamin A deficiency prevalence increased with age. Serum 25-OH-D3 level was used to evaluate the Vitamin D status as follow: vitamin D deficiency, serum 25-OH-D_3_<48 nmol/L; vitamin D marginal deficiency, serum 25-OH-D_3_ within 48∼78 nmol/L. The sum of vitamin D deficiency and marginal deficiency was near 90%. In the groups of children aged 37–48 months, although the prevalence of marginal deficiency was significantly higher in boys than in girls and the deficiency prevalence was significantly lower in boys than girls, however, the sum of marginal and deficiencies were not significantly different between boys and girls. There was not significant gender difference in serum retinol and 25-OH-D_3_ levels in the other age groups.

**Table 3:** The levels of serum retinol and 25-OH-D_3_ and deficiency prevalence of vitamin A and vitamin D of children^1^.

Age (m)	Boys			Girls		
	Concentration	Marginal, %	Deficient, %	Concentration	Marginal, %	Deficient, %
Retinol (µmol/L)						
24–36	1.21±0.54^a^	17.1^a^	2.9^a^	1.34±0.66^a^	12.0^a^	12.0^a^
37–48	1.35±0.56^a^	12.2^a^	7.3^ab^	1.19±0.66^a^	23.1^a^	15.4^a^
49–60	1.01±0.37^a^	25.6^a^	18.0^b^	0.99±0.37^a^	23.3^a^	20.0^a^
25-OH-D_3_ (nmol/L)						
24–36	58.2±46.8^a^	29.4^a^	52.9^a^	70.5±87.9^a^	26.9^a^	57.7^a^
37–48	50.2±21.8^a^	45.2^ab,^ 2	50.0^a,^ 3	45.5±19.2^a^	18.5^a^	74.1^a^
49–60	42.6±18.2^a^	21.6^b^	75.7^b^	42.4±19.2^a^	26.7^a^	70.0^a^

1 The results were expressed as mean±SD;

2 Significantly different from girls in marginal deficiency at the same age: *P*<0.05;

3 Significantly different from girls in deficiency at the same age: *P*<0.05.

### The other micronutrient concentrations and deficiency prevalence of children

The average serum level and deficiency prevalence of zinc in different age group were shown in Table 4. Serum zinc could be used as indicator of nutrition status: normal serum zinc level, ≥16.9 µmol/L (110 µg/dl); marginal zinc deficient level, <16.9 µmol/L (110 µg/dl); Zn deficiency, <11.0 µmol/L (72 µg/dl). Mean serum zinc concentrations in surveyed children were 13.3±3.7 µmol/L. Total percentages of deficiency including marginal and deficiency were 65.5% without gender difference in serum zinc level

**Table 4:** Serum zinc concentration and deficiency prevalence of children^1^.

Age (m)	Boys				Girls			
	n	Concentration (µmol/L)	Marginal (%)	Deficient (%)	n	Concentration (µmol/L)	Marginal (%)	Deficient (%)
24–36	34	13.8±4.2	67.7	23.5	26	12.9±3.7	53.9	34.6
37–48	42	12.9±2.4	69.0	26.2	27	13.6±2.8	66.7	25.9
49–60	37	13.6±4.0	59.5	34.4	30	13.2±3.0	73.3	23.3

1 The results were expressed as mean±SD.

The level of serum folic acid and vitamin B_12_ and deficiency prevalence of children were shown in Table 5. The mean serum folate levels were 19.3±11.9 nmol/L without folate deficiency and gender difference in surveyed children using serum folic acid level >16.9 ng/ml as cut-off point. The mean serum vitamin B_12_ levels were 344.6±227.9 ng/L. Total percentages of marginal and deficiency were 8.6% and 10.6% and the prevalence of marginal deficiency were significantly higher in boys than in girls.

**Table 5:** Folic acid and vitamin B_12_ levels and deficiency prevalence of children^1^.

Age (m)	Boys					Girls				
	n	FA Conc. (nmol/L)	B_12_ Conc. (ng/ml)	B_12_-m (%)	B_12_-D (%)	n	FA Conc. (nmol/L)	CB_12_ conc. (ng/ml)	B_12_-m (%)	B_12_-D (%)
24–36	34	19.5±11.4	319.7±242.5	12.1	12.1	26	18.7±11.4	353.0±222.2	7.7	7.7
37–48	42	18.0±8.4	365.8±262.3	16.7	7.1	27	20.3±17.7	303.0±186.8	7.4	11.1
49–60	37	20.4±11.1	320.3±171.9	10.8	5.4	30	19.2±12.7	416.7±261.7	3.3	10.0

1 The results were expressed as mean±SD; Abbreviations in table mean FA conc., folic acid concentration; B_12_ Conc., vitamin B_12_ concentration; B_12_-m, vitamin B_12_ marginal deficiency; B_12_-D, vitamin B_12_ deficiency.

## Discussions

Children aged 1–5 years is in a time of rapid and dramatic postnatal brain development, including neural plasticity, fundamental acquisition of cognitive development related to working memory and attention and inhibitory control. This period is also a key transition time for the child how is transferred from a direct maternal nursing and caring/selection of diet-based nutrition to food selection more based on self-selection and self-gratification. Anemia and micronutrient deficiencies in this period have been showed to have serious implications for individuals and societies. However, about the study evaluating the role of micronutrients in growth, brain and mental development, there have been fewer published related studies in preschool children than in infants or school-aged children [6]. In an emergency as Earthquake in Wenchuan, on the one hand, poor food choices, decreased accessibility to certain foods, coupled with the lack of knowledge feeding children may limit the inclusion of micronutrient-rich foods in the children diets; and on the other, preschool children have particularly high nutrient requirements for growth and development so that these children would have a relatively higher risk suffered from micronutrient deficiencies [3].

It has been reported that in displaced and emergency-affected populations, the most common method assessing the overall nutritional status in most vulnerable populations is to weight and measure growth and nutrition status of children aged 6–59 months [10], [11]. Based on our results on growth and development status results after one year of the Earthquake, the average stunting (HAZ<2SD) and wasting (WHZ<2SD) were respectively 14.7% and 0.7% (Table 1). Present survey showed that stunting and underweight (except 24–36 months) prevalence was significantly higher than the result of rural children average of 2002 National Nutrition and Health Survey, and there was no difference in wasting prevalence between this survey and the 2002 National Data which could be due to the higher stunting prevalence in the surveyed sites [12]. These results indicated that during such emergency situations, a general food basket (500 g cereals per day) provided by the government could meet the needs for the basic energy and protein to prevent acute malnutrition such as wasting, however, it was difficult to meet the needs to a variety of micronutrients due to shortage of animal foods and complimentary foods fitting for elder infants and young children) in child's daily diets for most nutritionally vulnerable group which could lead to long-term adverse effect such as higher prevalence of stunting and anemia, and the other micronutrient deficiencies such as vitamin A, vitamin D, vitamin B_12_, and zinc. Such long-term effects of malnutrition during early life not only could affect the growth and development, but also deeply involve the further cognitive ability and increase the greater risk susceptible to the chronic non-communicable diseases in adulthood [13], [14].

Because the consumption of micronutrient - rich food has been reported to be not common and no children had received iron-containing micronutrient supplements or lipid-based nutrient supplements in these areas [3], [15], micronutrients such vitamin A and vitamin D, iron, zinc, vitamin B_12_ would be most likely to be deficiency in children. There are some evidences that these micronutrients have specific critical roles in children's growth and brain nutrition [3]–[5], [16]. Deficiencies of iron, zinc, and vitamin A, vitamin B_12_ are major concern among children globally [17], [18], for example, World Health Organization has listed deficiencies of iron, zinc and vitamin A among the top ten leading causes of death in developing countries. Even if those who are deficient do not display clear clinical symptoms their physical and cognitive development are likely to be impaired, especially for children [19]. There is ample evidence of a range of micronutrients could play very important roles in brain development including iron, zinc, vitamin B_12_, folate and vitamin A and vitamin D. There is ample data in developing countries that a micronutrient deficiency during critical periods of brain development has a lasting impact which could resulted in adverse effects on health, growth and development, morbidity, cognitive and motor development [20]–[22].

Our study has evaluated anemia prevalence, and status of vitamin A and vitamin D, iron and zinc, vitamin B_12_ and folic acid (Table 2–5). We found that anemia was more common in children, average prevalence of anemia was 17.3% and percentage of iron deficiency was 45.7% (Table 2), which indicated that iron deficiency in children was very common and could be main reason for nutritional anemia in children. The prevalence of vitamin A deficiency and marginal deficiency was 15.4% and 30.3%, respectively, which was much more prevalent than the data (13.6–15.5% of children aged 3–6 years) in rural areas reported from the 2002 National Data [12]. The sum of vitamin D deficiency and marginal deficiency was near 90% (Table 3), which showed that our children had much higher prevalence of vitamin D deficiency compared with the children in 2011 Hangzhou city of southeast China [23]. Total percentages of zinc deficiency including marginal and deficiency were 65.5% (Table 4), which indicated that our children was also much higher prevalent than in the children living in rural areas in Jiangsu Province, China [24]. The percentages of vitamin B_12_ marginal and deficiency were 8.6% and 10.6% (Table 5), which prevalence was consistent with results of preschool children from Chongqing city, China. Present results indicates that vitamin B_12_ deficiency is a moderate public health problem in this area [25]. Micronutrient deficiencies in these areas were very common and could have long-term effect on growth and development of children. For example, anemia and iron-deficiency have been recognized as a major public-health problem in the world (including China) for many years, however, there has been little progress towards improvement and the global prevalence of anemia remained unacceptably high. Vitamin A deficiency has long been identified as a serious public-health problem in China [12], this vitamin is associated with blindness and with increased mortality and morbidity from severe infectious diseases. Vitamin D deficiency in children has been linked to adverse effects such as growth failure and rickets. The prevalence of vitamin D deficiency among children was between 12 and 24 percent [26]. The folate and vitamin B_12_ could play vital roles in the development of the brain, nervous system, and blood-forming organs and thus are essential for optimal growth and development in children. Improvement of micronutrient status of children in these emergency areas should be a priority urgently to be considered and solved.

### Limitations

The current results reported would here have certain limitations. In these surveyed areas, we could not recruit so many children in much decentralized and inaccessible areas and also we were difficult to find the basic data on growth and nutritional status and food supply or consumption in infants and young children before the earthquake because the national or regional nutrition surveys had not reach or cover these areas in the past. For those reasons, we have used the National Data and WHO grow curve standard for the comparison to show the severity of micronutrient deficiencies in these areas.

In summary, after an emergency as Earthquake, poor food choices, decreased accessibility to certain foods, coupled with the lack of knowledge feeding children may limit the inclusion of micronutrient-rich foods in the children diets so that these children would have higher stunting prevalence (long-term malnutrition) than the result of rural children average of 2002 National Nutrition and a relatively higher prevalence suffered from micronutrient deficiencies, including iron-deficiency anemia, and deficiencies of iron, zinc, and vitamin A and vitamin B_12_. Improvement of micronutrient status of children in these areas should be a priority. Providing the complementary food supplements and fortified foods would be the best and relatively mature way to combat the micronutrient deficiencies, and also publicly campaign how to promote the breastfeeding could be benefit in the nutritional improvement for local infants and young children.

## References

[1] Nishikiori N, Abe T, Costa DGM, Dharmaratne SD, Kunii O, et al (2006) Who died as a result of the tsunami?-Risk factors of mortality among internally displaced persons in Sri Lanka: a retrospective cohort analysis. BMC Public Health 6: 73–80. 16545145 10.1186/1471-2458-6-73 PMC1435747

[2] Wang LJ, Huo JS, Sun J, Li WX, Huang CY, et al (2010) Nutrition status of children aged 6–23 months living in Beichuan and Lixian effected by earthquake in Sichuan province. Chin J Prev Med 44 8: 696–670 (in Chinese) 21055018

[3] Zhao XF, Yin SA, Zhao LY, Fu P, Zhang J, et al (2010) The nutritional status among children under 60 months year-old after one year of the Earthquake in Wenchuan. Chin J Prev Med 44 8: 691–695 (in Chinese) 21055017

[4] Murphy SP, Allen LH (2003) Nutritional importance of animal source foods. J Nutr 133: S3932–5. 10.1093/jn/133.11.3932S 14672292

[5] Rosales FJ, Zeisel SH (2008) Perspectives from the symposium, The Role of Nutrition in Infant and Toddler Brain and Behavioral Development. Nutr Neurosci 11 3: 135–143. 18616870 10.1179/147683008X301522 PMC2562682

[6] Rosales FJ, Reznick JS, Zeisel SH (2009) Understanding the Role of Nutrition in the Brain & Behavioral Development of Toddlers and Preschool Children: Identifying and Overcoming Methodological Barriers. Nutr Neurosci 12 5: 190–202. 19761650 10.1179/147683009X423454 PMC2776771

[7] Black MM (2008) Effects of vitamin B_12_ and folate deficiency on brain development in children. Food Nutr Bull 29 2 Suppl: S126–S131. 18709887 10.1177/15648265080292S117 PMC3137939

[8] Benton D (2010) The influence of dietary status on the cognitive performance of children. Mol Nutr Food Res 54: 457–470. 20077417 10.1002/mnfr.200900158

[9] Wang Z, Yin S, Zhao X, Russell RM, Tang G, et al (2004) beta-Carotene-vitamin A equivalence in Chinese adults assessed by an isotope dilution technique. Br J Nutr 91: 121–131. 14748945 10.1079/bjn20031030

[10] WHO (1995) Physical status: the use and interpretation of anthropometry. Report of a WHO Expert Committee. Geneva: World health Organization. 8594834

[11] WHO (2000) The Management of nutrition in major emergencies, 2^nd^ ed, p236. Geneva: World health Organization.

[12] Yin SA, Lai JQ (2008) Nutrition and health status on 0 6 years of child in China. The nutrition and health status of Chinese children – 2002 China Nutrition and Health Survey. Beijing: People's Health Publishing House (in Chinese).

[13] Huang C, Li Z, Wang M, Martorell R (2010) Early life exposure to the 1959–1961 Chinese famine has long-term health consequences. J Nutr 140: 1874–1878. 20702751 10.3945/jn.110.121293

[14] Victora CG, Adair L, Fall C, Hallal PC, Martorell R, et al (2008) for the Maternal and Child Undernutrition Study Group (2008) Maternal and child undernutrition: consequences for adult health and human capital. Lancet 371: 340–357. 18206223 10.1016/S0140-6736(07)61692-4 PMC2258311

[15] Yin SA, Dong CX (2011) The nutritional status and improving ways of reproductive women and children in the disaster areas at about one year after Wenchuan Earthquake. Vitamins 85 suppl.: 97–111 (in Japanese)

[16] Young H, Jaspars S (1995) Nutrition assessments, food security and Famine. Disasters 19: 26–36. 7735853 10.1111/j.1467-7717.1995.tb00330.x

[17] Bwibo NO, Neumann CG (2003) The need for animal source foods by Kenyan children. J Nutr 133: 3936S–40S. 14672293 10.1093/jn/133.11.3936S

[18] Krebs NF (2007) Food choices to meet nutritional needs of breast-fed infants and toddlers on mixed diets. J Nutr 137: 511S–7S. 17237338 10.1093/jn/137.2.511S

[19] WHO (2002) The World Health Report, Geneva.

[20] Singh M (2004) Role of micronutrients for physical growth and mental development. Indian J Pediatr 71: 59–62. 14979388 10.1007/BF02725658

[21] Pelletier DL, Frongillo EA (2003) Changes in child survival are strongly associated with changes in malnutrition in developing countries. J Nutr 133: 107–19. 12514277 10.1093/jn/133.1.107

[22] Bryan J, Osendarp S, Hughes D, Calvaresi E, Baghurst K, et al (2004) Nutrients for cognitive development in school-aged children. Nutr Rev 62: 295–306. 15478684 10.1111/j.1753-4887.2004.tb00055.x

[23] Zhu Z, Zhan J, Shao J, Chen W, Chen L, et al (2012) High prevalence of vitamin D deficiency among children aged 1 month to 16 years in Hangzhou, China. BMC Public Health 12: 126. 22330045 10.1186/1471-2458-12-126 PMC3312872

[24] Qin Y, Alida MB, Zhao J, Wu M, Hu X, et al (2008) Stunting and zinc deficiency among primary school children in rural areas with low soil zinc concentrations in Jiangsu Province, China. Asia J Pac Clin Nutr 18: 15–21. 19329390

[25] Gao MZ, Li HQ (2006) Vitamin B12 nutritional status in preschool children in Chongqing. Zhonghua Er Ke Za Zhi 44: 7–10. 16623996

[26] Gordon CM, Feldman HA, Sinclair L, Williams AL, Kleinman PK, et al (2008) Prevalence of vitamin D deficiency among healthy infants and toddlers. Arch Pediatr Adolesc Med 162 6: 505–512. 18524739 10.1001/archpedi.162.6.505 PMC3206624

